# Enhancing Adolescent Girls’ Well-Being in the Arctic—Finding What Motivates Spending Time in Nature

**DOI:** 10.3390/ijerph18042052

**Published:** 2021-02-19

**Authors:** Varpu Wiens, Kari Soronen, Helvi Kyngäs, Tarja Pölkki

**Affiliations:** 1 Research Unit of Nursing Science and Health Management, University of Oulu, 90014 Oulu, Finland; helvi.kyngas@oulu.fi (H.K.); tarja.polkki@oulu.fi (T.P.); 2 Faculty of Social Sciences, University of Lapland, 96101 Rovaniemi, Finland; kari.soronen@ulapland.fi; 3 Oulu University Hospital, 90220 Oulu, Finland

**Keywords:** well-being, motivation, nature, adolescent, arctic, qualitative, second analysis, adolescent girls

## Abstract

Background: According to previous studies, the natural environment positively influences well-being, including that of adolescent girls. However, knowledge is lacking on what motivates adolescent girls to spend time in nature. A secondary analysis of qualitative data was conducted employing three preexisting sets of interview data that had formed the basis of previously published research reports. A novel perspective on what motivates adolescent girls in the Arctic to spend time in nature was uncovered—a finding that previous articles have not reported. Aim: The aim was to describe what motivates adolescent girls in the Arctic to spend time in nature. Methods: The participants were adolescent girls aged 13 to 16 living in the province of Finnish Lapland. The girls wrote about well-being (*n* = 117) and were interviewed (*n* = 19) about the meaning of seasonal changes, nature and animals’ influence on well-being. Also, five focus group interviews (*n* = 17) were held. The materials were analyzed by inductive content analysis. Results: After the secondary analysis, three generic categories were found: (1) wanting to have pleasant emotions, (2) the possibility of participating in activities and (3) a desire to feel better. The main category of “need to experience positive sensations” was formed. Conclusion: Based on these results, through personalized guidance and advice, it is possible to strengthen adolescent girls’ willingness to spend time in nature.

## 1. Introduction

According to previous studies, the natural environment and seasonal variation influence well-being. The effects of being in a natural environment on one’s well-being are wide-ranging. Such effects that were found in previous studies are restorative effects on physical and psychological well-being and impact different inflammatory diseases or psychiatric disorders [1]. In natural surroundings, it can be possible to lower one’s physiological arousal, which is a common condition with stressed individuals [2,3].

The importance of adolescents’ relationship with nature on their well-being has been studied since the 1990s [4,5]. Urbanization’s detrimental effects on young men’s experienced nature relatedness have been studied, with the assumption that in urban areas, action in natural environments is declining and being indoors is increasing [6]. A study compared natural environments’ influences on the well-being of adolescents versus other age groups [7]. Previous studies on young adults and adolescents have assessed natural environments as being more restorative than built ones [8,9]. One’s own experience plays an essential role in assessing a natural environment’s influence [10].

Natural environments’ influences on well-being have been studied widely since the 1980s. Since then, two main interpretations of the factors for the perceived improvements in well-being through being in a natural environment have had significant influences on further research. These main interpretations are stress recovery theory [11,12] and attention restoration theory [13,14]. Stress recovery theory’s main conclusion is that being in a natural environment can help one achieve calm emotional states and recover from stress-related physical symptoms [15]. Restorative influences of nature involve a shift towards a more positive emotional state and positive changes in physiological activity levels [12]. Studies have shown that heart rates and blood pressure levels drop in natural settings. In addition, excessive muscle tension is relieved after spending just a few minutes in a natural environment [16]. Regarding physical effects such as blood pressure and cortisol concentrations, research results are contradictory, and it is more difficult to obtain evidence of natural environments’ positive influence [17]. For the most part, recent empirical studies have provided significant support for stress recovery theory [18]. According to attention restoration theory, the recovery of attentional resources requires a calming natural environment [14]. Natural environments have plentiful characteristics necessary for restorative experiences [14]. It is essential that fascination and the restoration of attention can occur effortlessly [13]. Forests are estimated to help with recovery better than urban environments can because of the silence in forests and the presence of fewer people [19,20,21]. The natural environment has effects on mental well-being, such as restoring concentration and strengthening the experience of presence [22].

The natural environment’s importance to physical well-being has also been studied intensively. Growing up in a natural environment can reduce children’s atopic skin symptoms [23]. Researchers have become concerned about the link between biodiversity loss and the prevalence of inflammatory diseases. Different inflammatory conditions can affect the development of asthma, allergic symptoms and autoimmune diseases [24,25]. Recently, the prevalence of inflammatory diseases and microbial loss have been combined with higher incidence of depression [26]. According to several studies, mental health rehabilitees have benefited from being and acting in a natural environment. Growing up in a natural environment can lower the risks of psychiatric disorders [27], and natural surroundings are associated with lower schizophrenia rates among children and adolescents [28]. Adolescence is a time of growth and potential characterized by strong developmental events in the central nervous system; physical growth and development; hormonal changes; and changes in changes in emotions, thinking, behavior and interpersonal relationships [1]. These factors offer a reason to provide adolescents with support so that they can reach their full and healthy potential.

In some studies, researchers have compared exercising indoors with exercising outdoors in natural environments, as well as acting indoors with acting outdoors in built and natural environments [29]. The results indicated clearly greater positive influences for well-being combined with action in natural environments [30,31]. An interesting exception was feeling of calmness, which decreases when acting outdoors in a natural environment [32].

Finland is one of the northernmost countries in Europe and has a population density of two inhabitants per square kilometer. The country lies between the latitudes of 60° and 70° N and the longitudes of 20° and 32° E. In northern Finland’s natural environment, outdoor temperatures and the amount of daylight vary significantly by season. Cold weather in northern Finland usually begins in mid-October. North of the Arctic Circle, part of winter is the period known as the “polar night,” when the sun does not rise above the horizon. In the northernmost corner of Finland, the polar night lasts for 51 days. During winter, the mean temperature remains below 0 °C. The snow cover is deepest around mid-March, with an average of 60 to 90 cm of snow in northern Finland. Summer in northern Finland starts about one month later and ends a month earlier than it does on the southern coast. In summer, the mean daily temperature is consistently above 10 °C. However, warm weather in parts of northern Finland does not begin until June. The duration of a thermal summer in northern Finland is only two months or less in some places. The midnight sun is enjoyed in summer, mainly north of the Arctic Circle, when the sun does not set below the horizon. This time lasts 29–74 days in Lapland, depending on the location [33]. In northern Finland, natural features include vast areas of wild forest, open fells, and flora and fauna adapted to the harsh arctic conditions. Lapland has both forest-covered hills and open fells. In the far north, the tree line is so low that many fell-tops are treeless [34].

In addition to harsh weather conditions, distances and reduced service availability affect life in the Arctic. The influences of the natural environment and daylight on well-being mainly have been studied separately. Both seem to have parallel effects on well-being; therefore, interest in researching their common underlying mechanisms has increased [35,36]. Natural environments’ influences on the well-being of children, young people and especially adolescent girls [37,38,39,40] have received relatively little attention, most commonly from a mental health perspective [41,42].

On the whole, recent empirical research has corroborated growing evidence supporting natural environments’ beneficial implications for well-being. These implications also seem to be familiar to most people, so one would assume that this knowledge would motivate people to enjoy the benefits thereof. Yet what motivates people—and especially adolescent girls, presumably—is much more than just obtaining information and awareness. This motivation can be affected by social influences and perceived societal norms [43] or simply by the giving of choices [44].

Because motivation might be a need or a desire [45] that causes the individual to act, it is pivotal to know what conditions strived for cause adolescent girls to fulfill their motivation to spend time in nature. Following Ryan and Deci’s [46] conceptions, there is a need to know whether adolescent girls are motivated to spend time in nature because they are expecting external compensation or because they endorse the extrinsic goal’s value. On the other hand, motivation also has a context-specific dimension, and it arises from constant interactions between individual characteristics and the environment [47]. We know that adolescent girls in particular experience significant stress and anxiety [48,49], so the accepting presence associated with spending time in nature [50] offers girls an opportunity for integration. Because we know that nature relaxes and refreshes and that spending time in nature is beneficial, it is important to determine what motivates adolescent girls to spend time in nature, and adolescents’ own voices are still needed to articulate this.

In conclusion, the theoretical values of this research are based on stress recovery theory [11,12] and attention restoration theory [13,14], adolescence as a time of growth [35,36,41,42,50] and motivation [45,46,47]. Northern Finland’s habitat poses certain challenges to well-being, so it was relevant to focus specifically on the Arctic environment that had not been studied before. We had descriptions from the primary research of what affects adolescent girls’ well-being in the northern environment, and nature was perceived positively [37,38,39,40]. Therefore, this study’s research question is to determine what motivates adolescent girls to spend time in nature. The information produced by this research can be used to support the adolescent girls’ well-being and the development of a positive relatedness with nature.

## 2. Materials and Methods

### 2.1. Study Design

**Figure 1:**
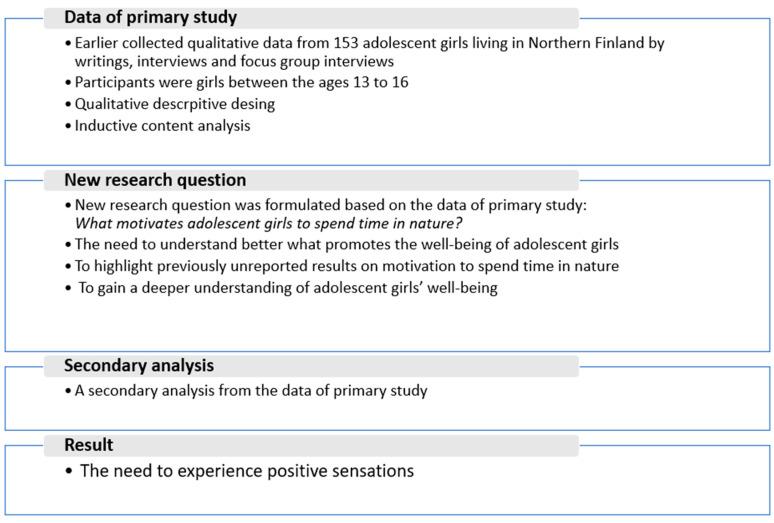
A secondary analysis of qualitative data from preexisting data of primary study.

A secondary analysis of qualitative data was conducted that employed three preexisting data sets (Figure 1). Secondary analysis was chosen for this descriptive work because it enabled us to address what had jointly emerged from the three data sets; specifically, it provided an opportunity to gain a deeper understanding of adolescent girls’ well-being and highlight previously unreported results on motivation to spend time in nature. As a result of the study, a new research question was formulated (Figure 1) on the basis of the data from the original, primary studies [37,38,39,40].

### 2.2. Participants

The participants were adolescent girls aged between 13 and 16 years old living in the province of Finnish Lapland, in northern Finland. Purposive sampling was used to ensure that a variety of girls with diverse backgrounds and perspectives participated in this research. The girls recruited for this research lived in northern Finland, which is located in the North Calotte and Barents region. Northern Finland has a variety of countryside settings and municipal centers as well as a few cities representing different identities, and it is characterized by a vast area and sparse population. To meet the inclusion criteria, girls had to be aged 13 to 16 years old in northern Finland and willing to participate in the research. The inclusion criteria did not take into account whether the participants were born in the region or from a different environment. Eventually, all of the adolescent girls who met the inclusion criteria and agreed to participate were included.

### 2.3. Data Collection

Because it is important to determine the purpose of the original research [51], it is described here (Table 1). In the original research, a descriptive qualitative design was used to elicit the girls’ perspectives through writings (*n* = 117), personal interviews (*n* = 19) and focus group interviews (*n* = 17) during which photographs were displayed. The same researcher (first author) collected the original data in each phase. In the first phase, written responses were chosen as a good data collection method with which to reach girls in a sparsely populated and vast region [52]. The data collection began after a pilot study was conducted in the spring of 2012 and after obtaining the necessary permits and consents. Instructions on how to conduct the study were sent in advance to the principals and teachers at the participating schools (Table 1). The study data, which consisted of the girls’ written responses [52], were submitted by computers and collected online without identifying data. The written material totaled 65 pages.

In the second phase, a semi-structured interview method was chosen to ensure that all required information was obtained and to provide the freedom for the girls to give as many illustrations and explanations as they wished [52]. The interviews were conducted between February and May 2014. Before the start of the study, a preliminary interview was carried out. Purposive sampling was used to enhance the information’s richness [52]. Information, instructions and consent forms were sent to the girls and their guardians in advance. On the appropriate date, the researcher traveled to the various parts of northern Finland, verified the signed consent forms and completed the interviews. Material was obtained from 19 girls aged 13–16 years old living in various parts of northern Finland. The total time of all interviews was 406 min 28 s. The duration of the interviews ranged from 10 min 17 s to 35 min 12 s. Interviews were conducted until data saturation was obtained.

In the third phase, focus groups were chosen as the data collection method because writings and individual interviews had been obtained earlier, and deeper knowledge and group perspectives on the phenomena were needed. One researcher oversaw five focus groups that each met once and were conducted between January and March 2015. Before the data collection started, a group of four 13- to 16-year-old girls living in northern Finland participated in a preliminary interview. Data collection started similarly to how it did in phase two. The girls were asked to participate in the focus group interviews and also to take photographs related to the research topic. There were 17 girls in total, with three girls each in four focus groups and five girls in one focus group. The total time for all of the interviews was 275 min (about 4.5 h), and the interviews lasted from 48 min to 1.5 h. During the focus group interviews, notes were made, and the interviews were recorded. The interviews were held until saturation was achieved. The girls presented 25 photographs of nature in total. The photographs’ content was discussed in the focus groups but was not analyzed.

The combined research data for the secondary analysis came from 65 pages of transcripts of the writings, 152 pages of transcripts from the personal interviews and 115 pages of transcripts from the focus group interviews conducted in 2012–2017 (Table 1).

The same researcher was responsible for data collection in the original research, primary analysis and secondary analysis. The focus group interviews were a continuation of previous phases of this research, which were individual interviews and writings. No moderator team was used, unlike what Krueger [53] suggested. According to Morgan [54] and Vaughn et al. [55], a well-designed focus group usually lasts between 1 and 2 h; in this study, the total time of all interviews was 275 min, and the duration of the interviews ranged from 48 min to 1 h 30 min.

### 2.4. Data Analysis

A secondary analysis of qualitative data was conducted that employed three preexisting interview data sets [56]. This provided an opportunity to increase the understanding of adolescent girls’ well-being. Inductive content analysis was chosen as the analysis method (Table 1). It was also the method used for primary analysis of the writings and the individual and focus group interviews because it is a systematic and objective means of describing and quantifying phenomena [57]. As Cavanagh [58] noted, the purpose of creating categories is to provide a means of describing a phenomenon to increase understanding and generate knowledge. The pilot interviews of the individuals and focus groups were not included in the analysis. The same researcher conducted the interviews and the transcription to avoid possible omission of words, misinterpretations or misspellings.

**Figure 2:**
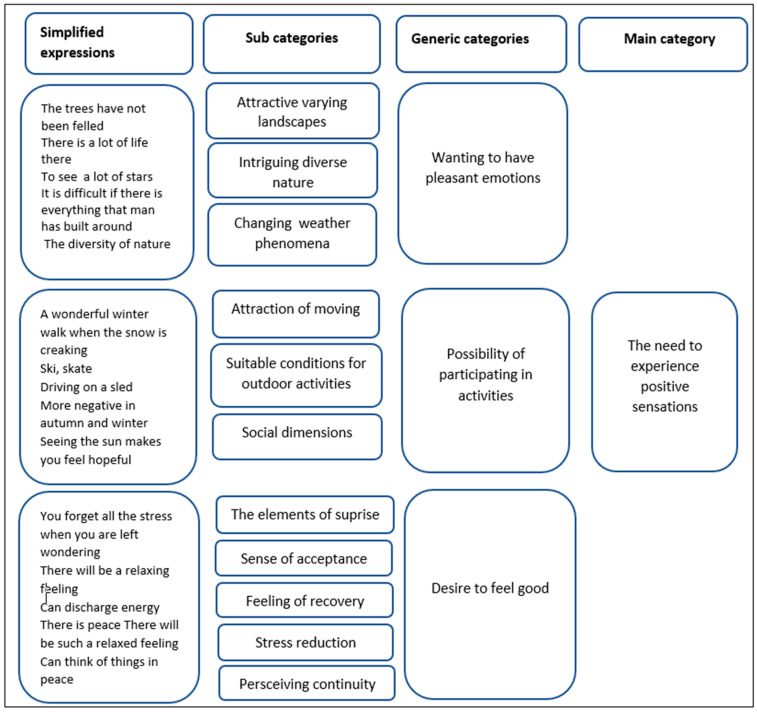
Illustration of the data analysis process showing the grouping of subcategories to form generic categories, and the main category: “the need to experience positive sensations.”

The secondary inductive analysis was guided by the research question of what motivates young girls to spend time in nature. In the secondary analysis, the writings and interviews were grouped according to themes, and within the themes, the analysis proceeded inductively using content analysis. In the first stage, each writing and interview was read from beginning to end several times to ensure that the researcher understood the overall contents. After the researcher became familiar with the data, the data organization began with coding. Notes and headings were written down and assigned to coding sheets. Words or statements related to the same central meaning were included in units of analysis [57]. The content was then reduced by converting the girls’ original expressions into simplified ones. Expressions that had similar meanings or dealt with related topics were grouped into subcategories, each of which was assigned a descriptive name. Subcategories with similar meanings were then grouped to form generic categories, which were named using content-characteristic words [57]. This abstraction process continued until new categories could no longer be formed and one main category was found (Figure 2). Although one researcher conducted the analysis, the categories were discussed and defined in the research group during the analysis process.

### 2.5. Ethical Considerations

Attention was paid to human dignity, which included participants’ consent and voluntariness, and to maintaining confidentiality throughout the study. The consent forms, information and instructions were sent in advance to the contact persons for them to hand to the girls and their guardians. These contact persons worked with young people in their own neighborhoods, such as youth workers. These contact persons gave information about the research to the girls. For the girls and their parents who gave consent to participate, a common interview date was agreed upon. Written consent was obtained from the participants and their parents, including for the usage of photographs. Before the interviews, each girl was again informed that she could withdraw from the study at any time if she wanted. The participants were assigned identification numbers to ensure their confidentiality so that their comments could not be linked back to their identities. Furthermore, the submissions were available only to the researchers. Before the interviews began, the girls were informed that the photographs would not be included in the analysis. Before the study began, it was approved by the Northern Ostrobothnia Hospital District Ethics Committee.

The study was also ethically justified because the data collection and transcripts of the primary data were rich and because it was reasonable to assume that the secondary research questions could be answered [59].

## 3. Results

Based on the secondary analysis of qualitative data of the adolescent girls’ descriptions of what motivated them to spend time in nature, the main category “need to experience positive sensations” was found (Figure 2). The three generic categories found were wanting to have pleasant emotions, the possibility of participating in activities and the desire to feel better.

### 3.1. Wanting to Have Pleasant Emotions

*“I like to lay on my back, look at the starry sky and let my thoughts go free.”*

The generic category of wanting to have pleasant emotions was formed from three subcategories: attractive, varying landscapes; intriguing, diverse nature; and changing weather phenomena. Attractive, varying landscapes motivate adolescent girls to observe visually beautiful scenery. The second subcategory of intriguing, diverse nature meant that adolescent girls observed different kinds of phenomena at both large and small scales. The totality of these sensations consisted of different sounds, colors, temperatures and lights. All of these phenomena, combined with the brightness and different compositions of the snow, aroused the girls’ interest in observing nature. As the girls moved through the woods, they noticed trees and their details, such as the greenery of the leaves or needles and the frosty branches. The girls described various water elements such as rivers and streams. The third subcategory of changing weather phenomena affected the girls’ motivation in different ways. The difficulty of finding suitable clothing decreased their motivation to spend time in nature, as did frostbite and feeling cold and wet. On the other hand, the girls appreciated the positive experience of the seasonal variations, and living in the north and having the opportunity to experience all four seasons motivated the girls to spend time in nature and monitor changes.

### 3.2. Possibility of Participating in Activities

*“In the winter, there are a lot of different things to do, like ice-skating and skiing.”*

The generic category of possibility of participating in activities was formed from three subcategories: the attraction of moving, suitable conditions for outdoor activities and social dimensions. The first subcategory of the attraction of moving means an attractive and liberating nature of moving, even spontaneously. In nature, one’s negative feelings can be resolved by talking aloud or even shouting. Being alone is an opportunity to sink into one’s own thoughts and “let go.” The girls reported not only walking but also sitting, lying down or jumping when moving in nature. Other physical activities included touching trees, plants and various materials or picking berries, mushrooms or cones. The second subcategory was suitable conditions for outdoor activities. Although the girls described wintertime as restrictive and said it reduced their motivation to spend time in nature, one of the positive aspects noted about it was snow, which, according to the girls, allows for winter sports such as skiing, ice-skating and snowmobiling. However, trying conditions noted were cold air and severe frosts, which are restrictive and reduce motivation. Ice prevented the girls from jogging, and snow made their clothes wet. However, getting out was effortless and motivating when there was light and warmth. The third subcategory was social dimensions: it was nice for the girls to spend time in nature and share experiences with friends. The social dimension did not always mean just human friends, but sometimes included also social connections felt with animals.

### 3.3. Desire to Feel Better

*“I just don’t know how it works. It, like, takes away all the troubles, and you can just let go. You do not need to worry about anything, just feeling good in beautiful surroundings.”*

The generic category of desire to feel better was formed from five subcategories: elements of surprise, sense of acceptance, feeling of recovery, stress reduction and perceived continuity. The first subcategory was the element of surprise the girls perceived when spending time in nature. As the girls conveyed, one never really knows what to expect, which has motivated them to spend time in nature. There are subtle, almost inexplicable things to discover, ranging from raindrops or snowflakes to more visible phenomena such as the aurora borealis or sunsets. The second subcategory was sense of acceptance, which the girls experienced when spending time in nature. The girls expounded that they could be themselves and did not have to think about how they looked or acted when spending time in nature. As one girl said, it felt liberating just being herself, without pressure. The third subcategory was feeling of recovery. Walking and wandering in nature resulted in the girls feeling empowered and invigorated. The fourth subcategory was stress reduction, of which there were rich descriptions. The girls were feeling stressed for various reasons, e.g., accumulating schoolwork or difficulties at home. As one girl explained, she would sometimes overcome all of her stress when she was just wondering at the versatility and formality of nature, which would make her forget everything in the moment. This state of relaxation and of feeling rejuvenated was an important perceived experience, as it gave strength to carry on with everyday life, and such states motivated the girls to experience them over and over again. The fifth subcategory was perceived continuity. When spending time in nature, the adolescent girls felt a sense of belonging and an experience of something greater.

### 3.4. Study Limitations

This research study had limitations related to sampling, data collection and the analytical process. The richness of the primary data is crucial, and so we have described the collection of such data. To ensure the richness and reliability of the primary data, they were collected in three phases, each time with the use of different qualitative methods to ensure saturation and comprehensive description of the phenomenon. One limitation involving sampling may be that the girls who were willing to participate in the study already perceived their health and well-being as good. One must also take into account that the study was conducted in a particular area, which affects the transferability of the results. Limitations on primary data collections were related to the writings, interviews and focus group interviews, specifically in the voluntary nature of the data collection process and the integrity of the written responses. It is possible that the girls felt obligated to answer because the written responses were gathered during the school day, and some girls might not have responded truthfully. Regarding the interviews, the first limitation is related to the sampling: although the 19 girls who participated in this study were from a wide range of different areas within northern Finland, the girls willing to participate in this study may have been those who already perceived their well-being as good. Regarding the focus group interviews, a moderator team was not used; instead, one person led the interviews and took notes. As described earlier in the data collection section, the total time of all interviews was 275 min, and the duration of the interviews ranged from 48 min to 90 min. This is in line with the recommendations [54,55]. It is also possible that focus groups may have prevented some adolescent girls from participating in the research. To ensure the intelligibility of the different data collection phases, a preliminary study was conducted each time.

The most commonly recognized limitation of the secondary data analysis method approach is “inherent in its nature” in that the data were collected for some other purpose [58]. The specific information that the researcher sought may not have been collected. The limitation related to primary data collection stems from the fact that although the researcher experienced a sense of data saturation after the interviews, more interviews could have been conducted to ensure this. The question arises as to whether the interviews reached the necessary depth to illuminate the meaning of different phenomena. In the primary data collection, participants were not directly asked whether they spend time in nature during their leisure time or, for instance, during schooldays. More emphasis was placed on descriptions of experiences, feelings and perceptions (Table 1). However, based on the girls’ answers, descriptions and photographs, we can assume that spending time in nature was undertaken in their leisure time. One limitation is that the girls took photographs at a particular moment in northern Finland for the researcher. However, in this secondary analysis, the researcher was the same one who collected the primary data and conducted the primary analysis and therefore had knowledge of the collection process and how it was conducted [59]. The earlier data provided an opportunity to gain a deeper understanding of well-being and to underline what motivates adolescent girls to spend time in nature. Therefore, secondary analysis of qualitative data was performed to complete the overall picture of well-being with a new research question [56].

The limitation related to the analytical process stems from the fact that one researcher conducted, audio-recorded and transcribed the interviews verbatim. This could pose a challenge due to the limitation of the researcher’s skills and experiences as well as the scope and sensitivity of the problem [55,57]. Another limitation is that nonverbal communication was not analyzed.

Because this was the secondary analysis, the researcher was already familiar with the data. To document the trustworthiness of the analytical process, some of the girls’ original sentences are presented in the text after having been translated from Finnish into English. Although some of the girls’ original sentences are presented in the text, some of the subtle meanings of the original statements may have been lost during the translation process. For the purpose of strengthening the credibility, the findings were discussed within the research group [55] and within a small group of five adolescent girls. In this second analysis, two rounds of inductive content analysis were performed, and both of these yielded similar generic categories.

## 4. Discussion

This study produced new knowledge, which is that the need to experience positive sensations motivates adolescent girls to spend time in nature. Positive sensations consist of wanting to have pleasant emotions, the possibility of participating in activities and the desire to feel better. These emotions and feelings were already explored in previous studies but have not necessarily been identified as a source of motivation. A comparison of this particular topic to other locations was difficult to make as the focus of this research was on the northern region, and no previous research on this topic could be found from other locations.

When the results of this study were compared with the Kaplan and Kaplan [13] elements, they were similar in terms of the desire for pleasant emotions, fascination, a sense of being away, the extent or scope of being a part of a larger whole and compatibility with an individual’s inclinations. When one looks at what issues motivate adolescent girls, the results would seem to suggest that the strongest motivation to spend time in nature is an internal need—the desire to have pleasant emotions—rather than external factors (e.g., social peer pressure or norms). The results of this second analysis are also reflected in the four channels of human interactions with the ecosystem [60]. Motivation can be found in all of the channels, which are knowing, perceiving, interacting and living. However, the clearest channel is the interacting one, which features physical, active and multisensory interactions with ecosystem components.

Based on the girls’ answers, one motivational issue is the possibility of participating in activities. Motivation can be increased when adolescents are given opportunities to make decisions and choices. Giving adolescents the autonomy and opportunity to follow their own interests when doing activities in nature could be enjoyable and rewarding for them. However, to achieve this, support and guidance are needed. We need to nourish adolescents’ connection to nature so that they do not lose the ability to interpret nature [61]. Girls could choose to go out on the basis of weather conditions or activities, or they could choose whether to ski, walk or use a snowmobile. One finding in this study was that snow had both negative and positive meanings for the girls. This view is supported by studies that have shown forest recreation in the snow during winter could increase psychological relaxation in females [62] and that snow might influence participants as a calming and emotion-lowering component of the environment. However, snow cover could be a slightly restraining factor in the environment because it halts the effect of the greenery on one’s vigor and vitality [63].

Measuring and monitoring one’s own results and changes can potentially increase motivation for adolescents to spend time in nature. According to Salonen [64], guiding people in recognizing the emotions generated from spending time in nature will help them to identify these emotions in the future as well. Along with friends, animals could also motivate girls to spend time in nature. Some concerns regarding gender equity in the outdoors [65] continue to be raised, but this issue did not come up in this research study.

A particularly important result from this research is the desire to feel better. This motivation to reduce stress levels is pivotal for adolescent girls. Girls view nature as common and equal to all; no criticism or judgment exists, and in nature they can be true to themselves. The feeling of stress relief is motivational as well. If spending time in nature supports the well-being of adolescent girls in a preventive way, it is significant in every aspect. Still, although all forests are restorative, some differences do exist, and it is important to preserve also old trees close to residential areas [66].

**Figure 3:**
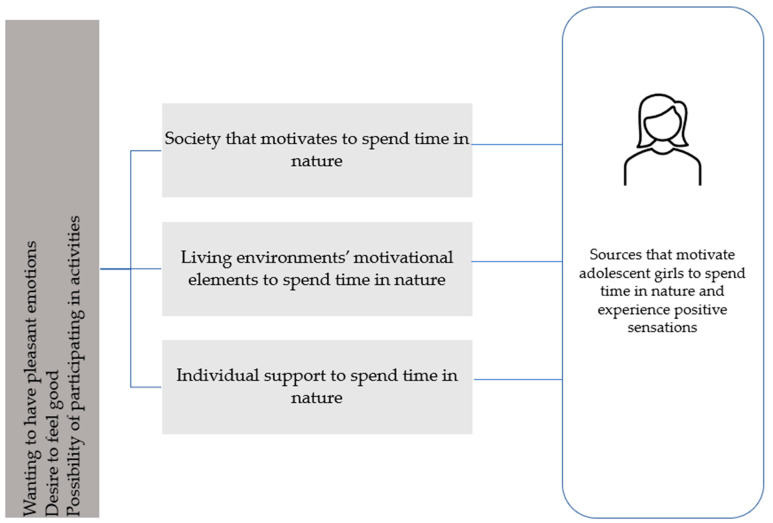
Summary of motivational elements and how to motivate adolescent girls to spend time in nature.

From the girls’ answers, one can conclude that they endorse the values of the internal goal. Their motivation also had a context-specific dimension and seemed to arise from the interaction between girls’ individual characteristics and the Arctic environment and nature. Now that we know what the motivational factors are, it is important that we identify these internal needs in adolescent girls. From previous studies, we know that adolescent girls experience symptoms such as stress, anxiety and depression. Thus, we can encourage and advise them to go out, enjoy nature and in turn improve their well-being. It is also worth noting that negative experiences can limit the motivation to spend time in nature [67]. In this research, participants mentioned dissatisfaction with unpleasant weather conditions, uncomfortable clothing and insects. These external issues can also be addressed in different ways in Arctic regions—for example, by equipping girls properly (in terms of clothing) or encouraging them to go out even in bad weather conditions. It is also possible to discuss with the girls or their families what missing clothes or equipment prevent them from spending time in nature. In addition, by spending time in nature together, the difficulties girls may face can be observed. If it is financially difficult for girls to procure equipment, various ways exist to help them. By responding to the need for motivation appropriately, we can support adolescent girls spending time in nature (Figure 3). Perceiving and experiencing well-being was reported widely and extended beyond girls themselves; it was affected by the girls’ environment, occurrences, incidences and happenings therein and social relationships. But as Caloqiuri and Chroni [68] state, the availability of a natural environment and attractive views of nature within an individual’s living environment are important contributors to physical activity.

Some evidence [69] indicates that adolescents take a “timeout” from preferring natural environments. Because this study was cross-sectional, we cannot confirm if this phenomenon was valid among adolescent girls living in the Arctic regions. The question is as follows: if this “timeout” shifts to a lower and by nature temporary preference in adolescents, should more emphasis be placed on this relatively brief duration? According to Kaplan and Kaplan [69], this phenomenon appears to occur during the period when children begin to prepare to take on adult roles. During this period, adolescents could use support in reinforcing their mental health [70]. In light of these results and earlier research findings, it is important to endorse contact with nature as it not only enhances well-being [71], but also may have effects on immune regulation [72] and intelligence [73].

Attempts can be made to strengthen and encourage adolescent girls’ motivation to spend time in nature by guiding them in this area. Because families play an important role in how children spend time in nature [74], family members should be involved in various ways by providing information and practical suggestions. Salonen [64] specifically emphasized that guiding methods can increase motivation because it can help reduce negative experiences and increase interest, which in turn increases the motivation to spend time in nature. When we encourage adolescent girls to spend time in nature, we also help them experience self-acceptance [75]. The assumption is that motivation also increases perseverance [76]. It is important to identify how outdoor recreations are presented [77] and whether they also allow intrinsic motivation [78] because adolescents are motivated by the wish to seek escape and to reflect [79]. Providing solitary time [80] would be one way to answer what girls seem to need, which also accords with the results of this research. Additionally, since the motivations might differ between genders [77], an individual approach is relevant by giving autonomy for decision-making [78]. The results of this study highlight the need for recovery and the need to experience positive sensations. Morse et al. [81] found in their research that there were significant increases in outdoor activity during the COVID-19 pandemic, especially among women. They discuss whether women may have had a greater need for stress relief during the pandemic and whether they are potentially more likely than men to turn to nature for stress relief. Recent research from northern Finland [82] concluded that residential greenness was positively associated with light physical activity in both genders, but after adjustments, the association remained significant only in men. This raises the question of whether a physical activity is the distinguishing factor here. This would reinforce the notion also found in this research that adolescent girls feel the need for stress relief and having positive sensations. On the basis of this study, it seems that girls have needs that motivate them to act to achieve balance. Girls’ needs to experience positive sensations are confluent with existence, relatedness and growth [83].

In this secondary analysis of qualitative data, we found the motivational elements for adolescent girls to spend time in nature through inductive content analysis. In the next phase, a hypothesis about the factors explaining the motivation will be formulated on the basis of these data and tested with statistical methods.

## 5. Conclusions

This secondary analysis was carried out to gain more and deeper understanding of how the well-being of adolescent girls living in northern Finland could be promoted. From previous studies, we know that nature has positive effects on well-being. To find out what motivates adolescent girls to spend time in nature, we did a secondary analysis to our original research with a new research question. Based on the results of this study, the need to experience positive emotions is what motivates adolescent girls to spend time in nature. Next, we need to consider how this motivation can be supported. We have outlined three different levels for recommendations (Figure 3). Adolescent girls are at the center of each recommendation. Each one of them lives and experiences a unique life, and adolescents should be consulted in all matters concerning them to identify external and internal individual motivations. In addition, here are recommendations of the enabling sources that should be considered. When giving individual support for spending time in nature, adolescent girls need encouragement, support and guidance. Girls could go alone, with a friend, or with a pet, according to one’s needs. They should be given opportunities to choose activities and provided with appropriate clothing and equipment. Because family is important to adolescent girls, spending time in nature should involve their families. Not only would this strengthen their relationship with nature, but also their relationships with family members and friends.

Living environments present various motivational elements to spend time in nature. In schools, students and their parents should receive more information about nature and their ability to use it. Local leisure activities and the design of the living environment play important roles in how accessible nature is. In community planning and decision making, nature and green areas must be made accessible and safe for girls. During schooldays, breaks should allow outdoor activities. Finland has a good school healthcare system, and knowledge about nature’s beneficial effects could be utilized when providing health education for adolescent girls.

A society that motivates people to spend time in nature has also a key role. Aspects of environmental well-being should be taken into consideration as part of health promotion programs for adolescent girls living in Arctic regions. These programs should provide means for them to facilitate connections with nature. In addition, in public health services, the role of nature’s influence on adolescent girls’ well-being should be acknowledged and utilized. Environmental protection involving nature preservation is important in northern environments, where nature recovers slowly from damage. National parks are available to everyone to enjoy, but they also have the crucial task of ensuring biodiversity. Part of this is good forest management and use. On a national level, it would be good to have guidance for communities on nature management and green zoning, as well as support for the management of health and well-being promotion in Finnish communities by giving recommendations taking into account nature’s effects on well-being.

Desiring positive feelings as well as opportunities to move outdoors is understandable during these uncertain times amid the pandemic. Thus, it is important to make sure that nature and green areas are accessible to and safe for girls. As we take care of nature, we also take care of our adolescents. However, as we encourage girls to spend time in nature, it is important that we keep the focus of our guidance flexible and youth-oriented so that we do not take away from the revitalizing effect of nature. When adolescent girls are motivated to spend time in nature, it offers benefits for the adolescents themselves and for nature in general. In nature, adolescent girls can take their mind off of everyday pressures and requirements and get in touch with their soul, inner thoughts and feelings.

## Figures and Tables

**Table 1:** The research questions in the original research and secondary analysis of qualitative data.

Methods	Themes	Analysis
Original Research		
Writings (2012–2013)	What is well-being to the girls as described by themselves? What things promote and hinder their well-being?	Inductive content analysis (65 pages of transcripts)
Personal interviews (2014)	What role do seasons play in the well-being of young girls? What role does nature play in the well-being of young girls?	Inductive content analysis (152 pages of transcripts)
Focus group interviews (2015)	What kind of experience do you have with nature, winter, and seasonal changes? How does nature, winter, and seasonal changes promote your well-being?	Inductive content analysis (115 pages of transcripts)
Secondary analysis (2020)	What motivates adolescent girls to spend time in nature?	Inductive content analysis (332 pages of transcripts)

## Data Availability

Data sharing not applicable due to the ethical issues agreed with the participants.
